# Chronic Immune Platelet Activation Is Followed by Platelet Refractoriness and Impaired Contractility

**DOI:** 10.3390/ijms23137336

**Published:** 2022-06-30

**Authors:** Izabella A. Andrianova, Alina I. Khabirova, Anastasia A. Ponomareva, Alina D. Peshkova, Natalia G. Evtugina, Giang Le Minh, Timur B. Sibgatullin, John W. Weisel, Rustem I. Litvinov

**Affiliations:** 1 Institute of Fundamental Medicine and Biology, Kazan Federal University, 420008 Kazan, Russia; izabella2d@gmail.com (I.A.A.); alina.urussu.95@gmail.com (A.I.K.); na.ponomareva@mail.ru (A.A.P.); alinapeshkova26@gmail.com (A.D.P.); natalja.evtugyna@gmail.com (N.G.E.); mrgiangleminh@gmail.com (G.L.M.); 2 Kazan Institute of Biochemistry and Biophysics, FRC Kazan Scientific Center of RAS, 420111 Kazan, Russia; 3 Department of Rheumatology, University Hospital, Kazan Federal University, 420097 Kazan, Russia; rustempa@gmail.com; 4 Department of Cell and Developmental Biology, School of Medicine, University of Pennsylvania, Philadelphia, PA 19104-6058, USA; weisel@pennmedicine.upenn.edu

**Keywords:** chronic immune inflammation, systemic lupus erythematosus, platelets, clot contraction, hemostatic disorders

## Abstract

Autoimmune diseases, including systemic lupus erythematosus (SLE), have a high risk of thrombotic and hemorrhagic complications associated with altered platelet functionality. We studied platelets from the blood of SLE patients and their reactivity. The surface expression of phosphatidylserine, P-selectin, and active integrin αIIbβ3 were measured using flow cytometry before and after platelet stimulation. Soluble P-selectin was measured in plasma. The kinetics of platelet-driven clot contraction was studied, as well as scanning and transmission electron microscopy of unstimulated platelets. Elevated levels of membrane-associated phosphatidylserine and platelet-attached and soluble P-selectin correlated directly with the titers of IgG, anti-dsDNA-antibodies, and circulating immune complexes. Morphologically, platelets in SLE lost their resting discoid shape, formed membrane protrusions and aggregates, and had a rough plasma membrane. The signs of platelet activation were associated paradoxically with reduced reactivity to a physiological stimulus and impaired contractility that revealed platelet exhaustion and refractoriness. Platelet activation has multiple pro-coagulant effects, and the inability to fully contract (retract) blood clots can be either a hemorrhagic or pro-thrombotic mechanism related to altered clot permeability, sensitivity of clots to fibrinolysis, obstructiveness, and embologenicity. Therefore, chronic immune platelet activation followed by secondary platelet dysfunction comprise an understudied pathogenic mechanism that supports hemostatic disorders in autoimmune diseases, such as SLE.

## 1. Introduction

Systemic lupus erythematosus (SLE) is a chronic autoimmune disorder, in which the immune system mistakenly attacks multiple organs and tissues of the body. Among many clinical features and complications, SLE is characterized by increased an risk of thrombosis and pulmonary hemorrhage, which are among the main causes of death [1,2,3]. Thrombosis in SLE has a prevalence that is more than 10% and may exceed 50% in the high-risk patients [4]. In addition to tissue damage and endothelial dysfunction, a possible reason for thrombosis in SLE is continuous systemic platelet activation by circulating immune complexes of various compositions [5,6]. In particular, the immune complexes formed by SLE-associated anti-dsDNA antibodies were previously shown to cause direct platelet activation in vitro [7]. Contrary to thrombosis, alveolar hemorrhage is a rare but dangerous complication of SLE with a prevalence of 2–5% [8] and a mortality rate of up to 92% [9]. The incidence and outcomes of alveolar hemorrhage in SLE patients are directly related to the disease severity as evaluated by the Systemic Lupus Erythematosus Disease Activity Index (SLEDAI) [10]. Some studies have pointed to a link between antiphospholipid syndrome (APS) and alveolar hemorrhage in SLE [8]. Although a history of thrombocytopenia is a strong predictor of alveolar hemorrhage in SLE, thrombocytopenia is not necessarily observed at the time of alveolar hemorrhage [8], suggesting that the alveolar hemorrhage is not caused by a low platelet count and must have a different mechanism, perhaps related to acquired thrombocytopathy.

Platelet activation, irrespective of a stimulus, is associated with surface expression and secretion of bioactive molecules, such as P-selectin, integrin αIIbβ3, and phosphatidylserine, which impart pro-coagulant and adhesive properties. In addition, these compounds can be used as molecular markers of platelet activation. 

Phosphatidylserines are negatively charged phospholipids located in the inner leaflet of the plasma membrane in resting platelets but may be externalized to the outer leaflet upon platelet activation. Activated platelets and platelet-derived microparticles with phosphatidylserine on their surface provide a matrix for the assembly and activation of clotting factors, thus promoting thrombin generation and hypercoagulability [11]. 

Integrin αIIbβ3 is an adhesive platelet surface receptor that is non-reactive in resting platelets but undergoes conformational activation when platelets are stimulated with a physiological trigger [12]. Activating events are associated with an increase in the affinity of the integrin αIIbβ3 for fibrinogen, which mediates platelet aggregation and platelet spreading on a fibrin(ogen)-containing matrix. Further, the interaction of αIIbβ3 with fibrin is involved in platelet-driven clot contraction [13].

P-selectin (CD62p) is another adhesive protein that is stored in the α-granules of resting platelets and becomes exposed on the platelet surface upon platelet activation by physiologic stimuli [14,15]. A fraction of surface-attached P-selectin can dissociate and circulate in the blood as a soluble protein named soluble P-selectin (sP-selectin). In addition to the sP-selectin in the blood that is shed from the platelet plasma membrane [15], a portion of secreted sP-selectin can originate from the alternative mRNA splicing that removes the exon encoding its transmembrane domain [16]. The amount of surface-bound P-selectin secreted from the platelet storage granules is counterbalanced by protein shedding, microvesiculation of the plasma membrane, and internalization [17].

Platelet-derived P-selectin (both membrane-associated and soluble) mediates binding interactions between platelets and other cells through its main ligand, P-selectin glycoprotein ligand-1 (PSGL-1), which is present mainly on the surface of leukocytes but also on dendritic and endothelial cells [18]. The binding interactions of the platelet P-selectin and PSGL-1 on leukocytes promotes inflammation through the enhanced production of TNF-α and interleukins (IL-1β, IL-6, IL-8, IL-12) [19]. At the same time, P-selectin-mediated interactions between platelets and leukocytes enhance the procoagulant activity of leukocytes by phosphatidylserine and tissue factor exposure [20]. The procoagulant potential can also be enhanced by the association of platelets with leukocyte-derived microparticles bearing a tissue factor [21].

The levels of platelet-associated and soluble P-selectins, as well as expression of platelet phosphatidylserine, were found to be elevated in the blood of patients with a number of prothrombotic conditions, such as venous thromboembolism and colorectal cancer [17,22]. Both forms of P-selectin, insoluble and soluble, were also overproduced during vascular damage and in patients with various cardiovascular diseases (acute myocardial infarction, stenotic coronary artery disease, and chronic heart failure), suggesting the association of P-selectins with such prothrombotic states [23,24]. It has also been shown that the fraction of platelets expressing P-selectin and phosphatidylserine is increased in SLE patients [25,26,27]. The data on the blood levels of soluble P-selectin (sP-selectin) in normal and disease states are controversial, and even in the plasma of healthy donors, the sP-selectin levels vary from an average of 24 ng/mL [28] up to 86 ng/mL [29], perhaps due to methodological variations [14,30]. To the best of our knowledge, the causative relation between the surface expressions of phosphatidylserine, P-selectin in resting platelets, the blood levels of sP-selectin, platelet morphological status, and reactivity in SLE has not been well studied.

Here, we tested a hypothesis that chronic continuous immune platelet activation followed by their secondary dysfunction, including impaired contractility, may have pro-thrombotic or hemorrhagic effects. To document platelet activation, we investigated platelet morphology as well as surface-associated phosphatidylserine, active integrin αIIbβ3, P-selectin, and soluble sP-selectin in correlation with the kinetics of blood clot contraction as an integral measure of platelet functionality. All the quantitative parameters were measured in parallel in the same blood samples of SLE patients. In addition, platelets from SLE patients were studied with scanning and transmission electron microscopy to assess the structural changes associated with platelet activation and their functional state. The results show that platelets circulating in the blood of SLE patients have multiple pronounced signs of activation that are paradoxically associated with reduced platelet contractility and reactivity as a result of platelet exhaustion. We propose that the continuous primary immune platelet activation and their secondary dysfunction, at least in a substantial fraction of platelets, may have both pro-thrombotic and hemorrhagic effects in SLE and probably in other chronic autoimmune diseases.

## 2. Results

### 2.1. Continuous Background Activation of Platelets in the Blood of SLE Patients

#### 2.1.1. Molecular Markers of Platelet Activation in SLE

**Figure 1:**
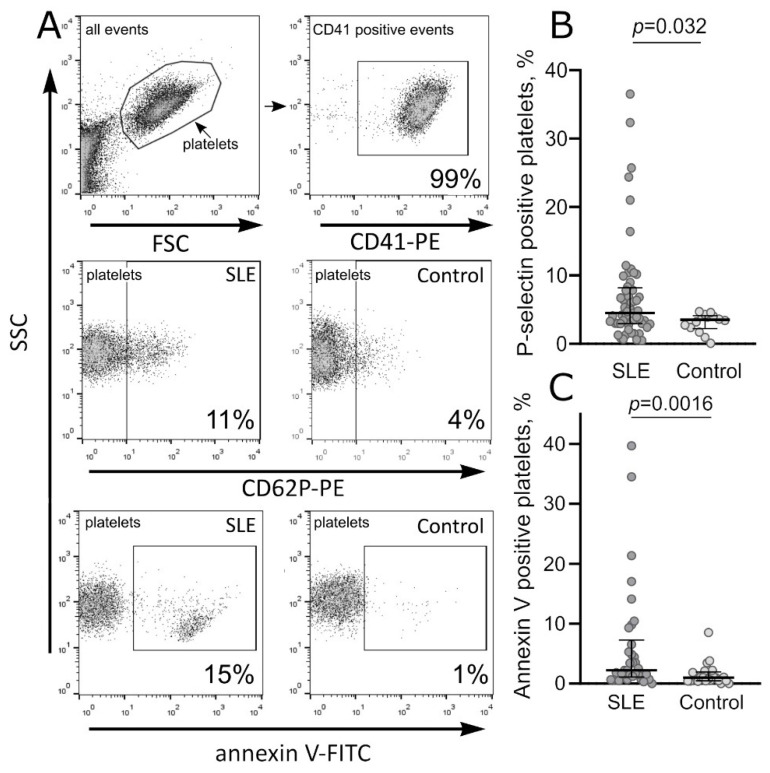
Flow-cytometry-based gating and quantification of platelets expressing P-selectin and phosphatidylserine. (**A**) *Upper row*: Representative dot-plots of platelets from the blood plasma showing the platelet gating strategy based on the forward (FSC) and side (SSC) scatter values (left) and verified using a platelet specific marker CD41 (right). *Middle row*: Representative dot-plots demonstrating the fractions of in vitro unstimulated CD62p-PE-positive platelets (expressing P-selectin) in the blood of an SLE patient (left) and a healthy donor (control). *Bottom row*: Representative dot plots showing the fractions of in vitro unstimulated annexin V-FITC-positive platelets (expressing phosphatidylserines) in the blood of an SLE patient (left) and a healthy donor (control). (**B**) Fractions of unstimulated platelets expressing P-selectin (CD62p) on the surface in the SLE patients (*n* = 56, dark grey dots) and healthy donors (control, *n* = 14, light grey dots) based on the gating strategy shown in A. (**C**) Fractions of unstimulated platelets expressing phosphatidylserine in the SLE patients (*n* = 34, dark grey dots) and healthy donors (control, *n* = 22, light grey dots) based on the gating strategy shown in A. The data in (**B**,**C**) are presented as a median with IQR and analyzed with a Mann–Whitney test.

Platelets isolated from the blood of SLE patients without additional stimulation had a 2-fold larger average fraction of platelets with detectable phosphatidylserine on their surface compared to the unstimulated in vitro platelets from healthy donors (Table 1a, Figure 1A,C). In particular, the fraction of phosphatidylserine-expressing platelets in the SLE patients varied from 0.03% to 40%, while in the control platelets from healthy subjects, the range was 0.005–8.5% (*p* = 0.0016), indicating chronic background platelet activation associated with SLE.

Unstimulated platelets from the blood of SLE patients also had a significantly larger fraction of P-selectin-positive platelets than platelets from the healthy donors (Table 1a, Figure 1A,B). Specifically, the fraction of P-selectin-positive platelets in the SLE patients ranged from 0.5% to 36.5%, while in the platelet preparations from healthy subjects, the range was 0.1–4.7% (*p* = 0.032). The group of SLE patients had an increased average level of sP-selectin in plasma (51 ± 18 ng/mL) compared with the normal values (34 ± 5 ng/mL, *p* < 0.001) as also reported by [14] (Table 1b). The hyper-production of P-selectin likewise indicates moderate continuous background platelet activation in SLE.

#### 2.1.2. Morphological Signs of Platelet Activation in SLE

**Figure 2:**
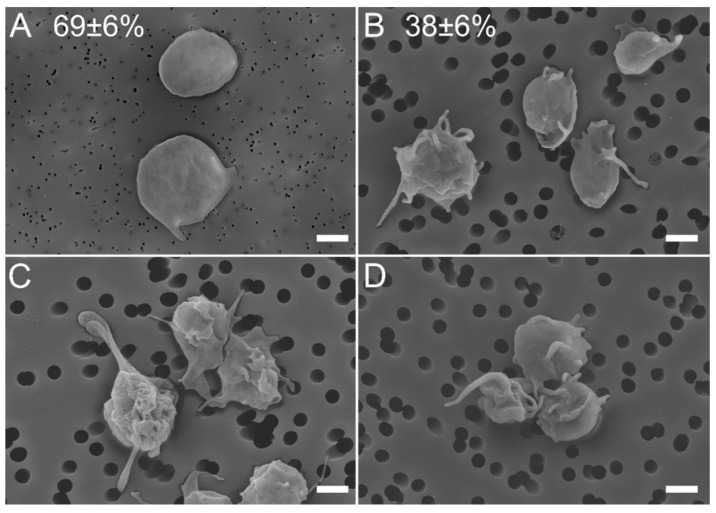
Representative scanning electron micrographs show a background activation of circulating platelets in the blood of SLE patients. (**A**) Characteristic morphology of unstimulated resting platelets isolated from the blood of a healthy subject (control). (**B**–**D**) Unstimulated platelets isolated from the blood of SLE patients with morphological signs of activation: loss of discoid shape, formation of filopodia and other membrane protrusions, and aggregation. Scale bars = 1 µm. Numbers represent the percent of platelets with the morphological signs of resting/quiescent state in control (**A**) and SLE (**B**) samples. Platelets were settled on the polycarbonate filter with 0.1 µm (**A**) or 0.4 µm (**B**–**D**) pore size.

Using scanning electron microscopy, we examined the morphology of 695 individual platelets isolated from the blood of four SLE patients and 662 platelets from four healthy donors. On average, only 38 ± 6% of platelets from SLE patients had the morphological characteristics of non-activated quiescent platelets with a discoid shape and smooth membrane, sometimes with 1–2 short filopodia (Figure 2A). In contrast, the quiescent platelets comprised 69 ± 6% in control samples from healthy subjects (*p* < 0.001, χ^2^-test). Most of the platelets in the SLE preparations (62 ± 6%) had various morphological signs of activation, namely multiple filopodia or lamellipodia and/or loss of their normal discoid shape, sometimes associated with the shrinkage of the platelet body, assessed as a decrease in the average platelet body diameter (Figure 2B,C). Some platelets in the SLE samples, unlike in the control, formed small aggregates (Figure 2D), which is another sign of platelet activation.

#### 2.1.3. Ultrastructural Alteration of the Platelet Plasma Membrane in SLE

**Figure 3:**
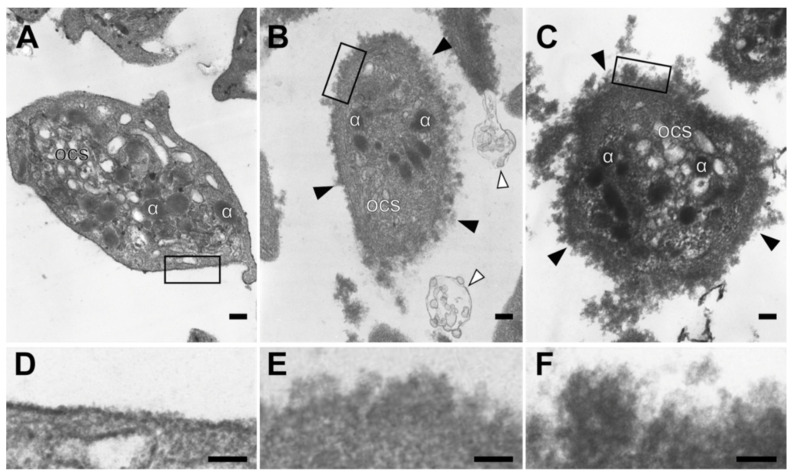
Representative transmission electron micrographs showing ultrastructural alterations of platelets in SLE. Platelets were isolated from the blood of a healthy donor (**A**) and SLE patients without (**B**) and with (**C**) the antiphospholipid syndrome, showing ultrastructural alterations of platelets in SLE. Black arrowheads indicate the altered plasma membrane, and white arrowheads indicate plasma-membrane-derived multivesicular structures. (**D**–**F**) Corresponding zoomed-in areas of interest from (**A**–**C**) are marked with a rectangle, illustrating an abnormally rough and shaggy platelet membrane in SLE. Designations: α—α-granules; OCS—open canalicular system. Scale bars = 200 nm (**A**–**C**) and 100 nm (**D**–**F**).

The ultrastructure of 94 individual platelets from six SLE patients and 60 cells from three healthy subjects were examined using transmission electron microscopy. The structure of the platelet plasma membrane was the most remarkable difference between control platelets and platelets from SLE patients. In the control platelets isolated from healthy subjects, the plasma membrane was smooth (Figure 3A), while many SLE platelets had an unusual shaggy and rough plasma membrane (Figure 3B,C), with alterations that were much more pronounced in the patients that had the antiphospholipid syndrome (Figure 3C,F). Platelets with altered/abnormal plasma membrane were observed in 100% of SLE patients studied. In addition, the platelet preparations from SLE patients often contained multi-vesicular particles, some of which were obviously derived from the platelet plasma membrane (Figure 3B). Otherwise, the platelets from both healthy subjects and SLE donors were 1–3 μm in size and had a similar discoid shape, sometimes with one or two short filopodia, but most of them had no membrane protrusions. Intracellular components (α-granules, dense granules, open canalicular system, lysosomes, and mitochondria) were somewhat less distinct in the platelets from SLE patients compared with normal platelets, but the overall structure of cytoplasm and intracellular organelles were essentially the same (Figure 3). The ultrastructural changes revealed in the plasma membrane of SLE platelets suggest the formation of multimolecular deposits on the platelet surface, likely comprising immune complexes interacting with the glycocalyx.

### 2.2. Impaired Functionality of Platelets in the Blood of SLE Patients

#### 2.2.1. Poor Reactivity of Platelets in Response to Stimulation in SLE

**Figure 4:**
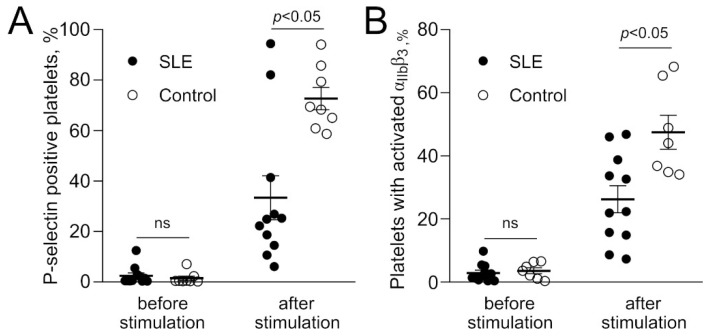
Differential reactivity of platelets isolated from the blood of SLE patients and healthy donors (control) in response to stimulation. Flow cytometry was used to measure a fraction of platelets expressing P-selectin (**A**) or active αIIbβ3 (**B**) before and after activation with 50 µM TRAP. Gel-filtered platelets were isolated from the blood of 11 active SLE patients (black dots) and 8 or 7 healthy subjects (white dots) for the measurements of the surface expression of P-selectin and active αIIbβ3, respectively. ns = not significant. Mann–Whitney test.

To quantify platelet reactivity, we measured the fraction of activated platelets before and after TRAP-induced stimulation of PAR1 receptors. Using flow cytometry, activated platelets were assessed as positive for P-selectin (Figure 4A) or bearing integrin αIIbβ3 in its active conformation on the surface (Figure 4B). While the initial expression levels of the activation markers on resting platelets were similar, after stimulation with TRAP, platelets from SLE patients had a significantly lower share of activated platelets compared with platelets from healthy donors (Figure 4). Specifically, in the platelets from SLE patients, the addition of TRAP increased the average fraction of P-selectin-positive platelets from 2.5% before to 33% after TRAP stimulation, while in control samples, the average fraction of platelets expressing P-selectin increased from 1.5% to 73% after TRAP stimulation. The average fraction of SLE platelets with activated αIIbβ3 after TRAP-induced stimulation increased from 2.9% to 26%, while in control platelet preparations, it increased from 1.5% to 48%. These results indicate substantially reduced platelet reactivity or partial platelet refractoriness in SLE.

#### 2.2.2. Decreased Platelet Contractility in SLE

**Figure 5:**
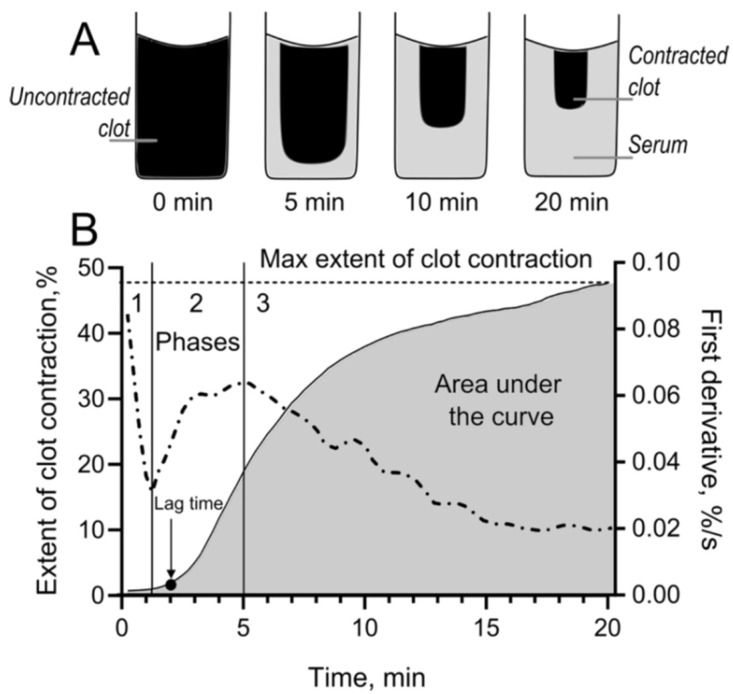
Kinetics of blood clot contraction measured using an optical tracking system. (**A**) Schematic illustration of the contracting clot size changing over time. (**B**) An output kinetic curve with the corresponding parameters of clot contraction: the maximum extent of clot contraction after 20 min, lag time, and area under the curve reflecting mechanical work performed by platelets. Using the local minima and maxima of the first derivative (dashed line), the clot contraction kinetics is segregated into three phases, corresponding to the initiation of contraction (phase 1), linear contraction (phase 2), and mechanical stabilization (phase 3) as suggested in [32].

Among a variety of platelet functions involved in the pathophysiology of hemostatic disorders, one of the least studied is platelet contractility, which is responsible for the mechanical remodeling and compaction of blood clots and thrombi, thus affecting their obstructiveness, embologenicity, permeability, susceptibility to fibrinolysis, and other biological properties [31]. Therefore, as an important integral characteristic of platelet functionality and responsiveness to stimulation, we measured the kinetics of blood clot contraction, where platelets are activated with exogenous thrombin (Figure 5). 

**Figure 6:**
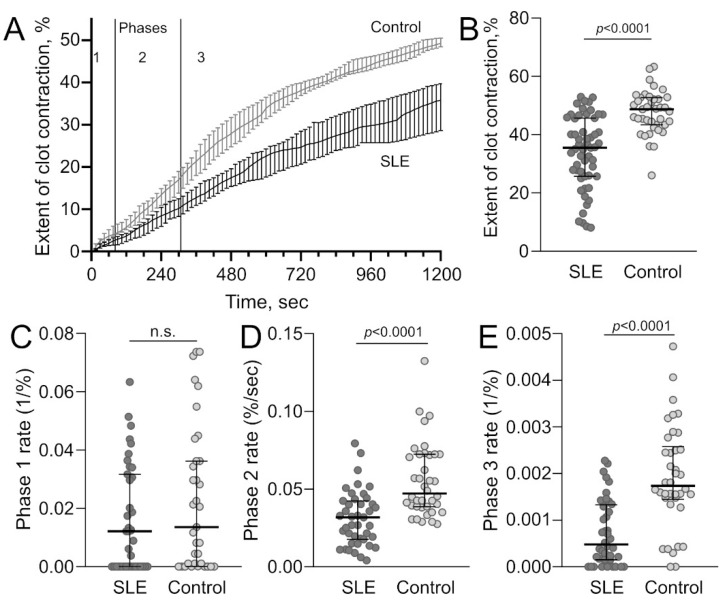
Kinetics of platelet-driven contraction of clots from the blood of SLE patients and healthy subjects. (**A**) Averaged kinetic curves of clot contraction (median and 95% confidence interval) in SLE patients (*n* = 55) and healthy subjects (control, *n* = 40). The borders between phases of contraction were determined by finding local minima and maxima of the instantaneous first derivative of the kinetic curves. (**B**) A decreased extent of clot contraction in SLE patients (dark grey dots) compared to healthy subjects (control, light grey dots). (**C**,**D**) Kinetic phase analysis of clot contraction showing no difference in the rate constants for phase 1 (**C**) and a significand decrease in the rate constants for phase 2 (**D**) and phase 3 (**E**) in the SLE patients (dark grey dots) compared to controls (light grey dots). The results in B-E are presented as the mean and IQR. n.s. = not significant. Mann–Whitney test.

In the blood of SLE patients, the average rate and extent of clot contraction were significantly impaired compared to healthy donors (Table 1c, Figure 6A). In particular, the median extent of contraction was reduced by 27% (Figure 6B), the lag period was prolonged about 2-fold, and the median area under the kinetic curve (mechanical work done by platelets) and velocity were decreased by 31% and 24%, respectively. 

It has been shown earlier [32] that blood clot contraction occurs in three phases: initiation of contraction (phase 1), linear contraction (phase 2), and mechanical stabilization (phase 3) (Figure 5B). The kinetic phase analysis revealed that in SLE, the rate constants of phases 2 and 3 of clot contraction were reduced 1.5- and 3.6-fold, respectively (Table 1d, Figure 6D,E), indicating the deceleration of the linear contraction and mechanical stabilization of clots in the blood of SLE patients. Hence, this impaired contractility is another sign of platelet dysfunction in SLE that may have important pathogenic consequences, as discussed below.

#### 2.2.3. Relationship between the Parameters of Platelet Activation, Contractility, and Immune Inflammation in SLE

To reveal the potential functional relationships between systemic inflammation and platelet functionality in SLE, we performed a comprehensive correlation analysis using the quantitative parameters obtained. The significant and meaningful correlation coefficients are presented in Table 2. 

*Expression of phosphatidylserine* (Table 2a). The percentage of platelets bearing phosphatidylserine on their surface had a negative correlation with the lag time of blood clot contraction (r = −0.39, *p* = 0.03), suggesting that the generation of endogenous thrombin on the procoagulant platelet membrane accelerates contraction. Additionally, the percentage of phosphatidylserine-expressing platelets had a positive correlation with the disease severity, measured as SLEDAI (r = 0.48, *p* = 0.012), which was even more pronounced in patients with APS (r = 0.84, *p* = 0.029), supporting the causative relationship between the systemic immune inflammation and platelet activation. The leukocyte counts correlated inversely with the fraction of phosphatidylserine-positive platelets, but the functional relation between these parameters is unclear. 

*Surface expression of P-selectin* (Table 2b). The expression levels of P-selectin on platelets correlated positively with the extent of clot contraction (r = 0.31, *p* = 0.029), confirming that in the fraction of activated platelets that still maintain functionality, the platelet contractility and granule secretion are concerted responses to the activation. The expression of platelet-associated P-selectin correlated directly with the markers of systemic immune inflammation in SLE. The fraction of P-selectin-positive platelets correlated directly with the titers of anti dsDNA antibodies (r = 0.46, *p* = 0.013) and total IgG levels (r = 0.33, *p* = 0.031). In support of this relationship, the SLE patients with elevated levels of circulating immune complexes in the blood (>120 U/mL) had a significantly increased percentage of P-selectin-positive platelets (6.8%) compared to the SLE patients with normal levels of circulating immune complexes (3.8%, *p* = 0.007, Mann–Whitney test). There were significant positive correlations between the percentage of P-selectin-bearing platelets and the blood levels of IgA and IgG (r = 0.69, *p* = 0.032, and r = 0.67, *p* = 0.039, respectively) for SLE patients with APS. Notably, SLE patients without a history of thrombosis had a positive correlation between the fraction of platelets with P-selectin and the disease severity as assessed by the SLEDAI score (r = 0.49, *p* = 0.001).

*sP-selectin* (Table 2c) levels in SLE patients correlated directly with the titers of anti-dsDNA antibodies (r = 0.43, *p* = 0.03) and with the blood levels of IgA (r = 0.35, *p* = 0.031) and IgM (r = 0.47, *p* = 0.003). Correlation between soluble and platelet-associated P-selectin was not observed (r = 0.12, *p* = 0.42), likely because sP-selectin, unlike platelet-associated P-selectin, was eliminated from the blood by leukocytes via PSGL-1-mediated interactions [33,34]. Remarkably, in the SLE patients with APS, the levels of sP-selectin correlated positively and strongly with the levels of IgM (r = 0.74, *p* = 0.04), IgA (r = 0.67, *p* = 0.04), and anti-dsDNA antibodies (r = 0.82, *p* = 0.034). Patients with a history of thrombotic events also showed strong positive correlations between the levels of sP-selectin and IgM (r = 0.94, *p* = 0.004) and anti-dsDNA antibodies (r = 0.99, *p* < 0.001).

## 3. Discussion

Activated platelets play a major role in thrombotic complications, and the background level of activated platelets is increased in patients with SLE, especially with high SLEDAI [35]. The continuous background platelet activation can be induced in SLE by various autoimmune complexes (via FcγRIIA receptors), by the complement system components (via receptors for C1q, C4, etc.) [36], and by tissue-damage-associated molecules, such as DNA- or RNA-containing complexes (via Toll-like receptors) [35]. Earlier, we showed that in SLE, platelets are activated by anti-dsDNA Abs alone or in a complex with dsDNA via the FcγRIIA receptor [7]. In this work, multiple signs of chronic platelet activation in the blood of SLE patients have been demonstrated, including the overexpression of phosphatidylserine and P-selectin and increased secretion of P-selectin, as well as characteristic shape changes and alteration of the plasma membrane (Figure 1, Figure 2 and Figure 6, Table 1a,b). Although activated endothelium can be an additional source of a relatively small fraction of sP-selectin, the main source of sP-selectin is platelets [37]. The hyper-production of P-selectin in SLE patients is associated with the elevated titers of anti-dsDNA antibodies, which are good indicators of the prothrombotic and inflammatory status in SLE associated with the immune activation of circulating platelets. An increased fraction of P-selectin-bearing platelets associated with an increased level of circulating immune complexes, as well as a positive correlation of platelet-associated and soluble P-selectin with the anti-dsDNA autoantibodies (Table 2b,c), supports the conclusion of continuous immune activation of platelets, which agrees with the literature [27]. Associations between (s)P-selectin and the markers of immune inflammation are especially pronounced for SLE patients with APS (Table 2b,c). Taken together, these and other results clearly show that platelets are highly activated in SLE and as such can play a strong prothrombotic role in autoimmune disorders.

Unlike the prothrombotic effects of immune platelet activation, the mechanisms of bleeding complications of SLE, such as pulmonary bleeding, are much less understood, including the unidentified role of acquired platelet abnormalities. According to the literature, alveolar hemorrhage may be associated with cardiac valve disease, C3 hypocomplementemia, serologically high titers of anti-dsDNA antibodies, leukopenia, etc. [8,9]. Importantly, most of the SLE patients with alveolar hemorrhage did not have thrombocytopenia but, paradoxically, might have signs of arterial thrombosis [8], which suggests that the alveolar hemorrhage may be related to the secondary platelet dysfunction following primary chronic background platelet activation. Based on the literature and our own observations [38,39,40,41], we hypothesized that after a period of augmented functionality induced by autoimmune complexes and other stimuli generated in SLE, platelets would become exhausted and dysfunctional. Our present findings support this hypothesis and shed light on the mechanisms underlying the activation-related secondary platelet disability. Using a combination of flow cytometry and biomechanical measurements, we showed that immune-induced platelet activation is followed by their dysfunction, which is manifested in two main signs. First, the SLE platelets were partially refractory; i.e., their functional response to biochemical stimulation was relatively weak and did not cause full surface expression of the molecular markers of platelet activation, such as P-selectin and the activated form of the integrin αIIbβ3 (Figure 4, Table 2c). Second, platelets from the blood of SLE patients had impaired contractility, which manifested as reduced and slowed blood clot compaction driven by platelet-generated traction and compressive forces (Figure 6, Table 1).

The paradoxical combination of the signs of platelet activation and dysfunction has at least three conceivable explanations. The most likely mechanism of the dysfunction following platelet activation is energetic exhaustion. The metabolic ATP reduction in chronically stimulated platelets may be due to progressive mitochondrial depolarization [42], as well as to impaired glycolysis, both important sources of ATP in activated platelets [43,44]. The insufficiency of ATP is aggravated by its consumption during energy-demanding platelet functions, such as contractility [42]. The second plausible mechanism of secondary platelet dysfunction is the acquired storage pool deficiency or storage lesions related to depletion of the content of the secretory granules in continuously activated or aged platelets [45]. The third possible explanation of platelet dysfunction is based on the shedding of various receptor molecules from the plasma membrane after chronic platelet activation. The shedding of surface receptors such as GPIbα, GPV, and GPVI, as well as P-selectin and CD40L, may lead to decreased platelet functionality due to the inhibition of receptor-mediated adhesive interactions [17]. Irrespective of the underlying mechanism(s), continuously activated platelets become exhausted, refractory, and have diversely impaired functionality. As a matter of fact, our findings are in agreement with an array of papers that describe the connection of platelet activation and subsequent dysfunction revealed in other pathological conditions, including a relatively new pathological condition known as vaccine-inducted thrombotic thrombocytopenia (VITT) [46,47,48,49,50,51,52,53].

In addition to the more obvious effects of platelet dysfunction, impaired clot contraction leads to increased permeability of clots and hence more bleeding [54]. The impaired platelet contractility as a potential prothrombotic mechanism is less apparent, although the inability of platelets to cause full clot contraction has been observed earlier in a number of (pro)thrombotic states of various origins [32,55,56,57]. The reduced ability of blood clots and thrombi to shrink can promote thrombosis via several pathogenic mechanisms that include increased obstructiveness of thrombi [56,57,58,59], reduced susceptibility to fibrinolysis [60], and increased embologenicity or reduced resistance to rupture [56,61]. Therefore, the secondary platelet dysfunction following platelet activation is a novel hemostatic mechanism in SLE and probably in other autoimmune disorders. 

In summary, we studied the functional state of platelets in the blood of SLE patients in relation to the prothrombotic or bleeding propensity observed in autoimmune disorders. The main finding is that platelets in the blood of SLE patients displayed functional and morphological signs of immune activation associated paradoxically with impaired platelet reactivity and contractility. Platelet activation is a well-known prothrombotic mechanism due to pro-coagulant and adhesive properties of activated platelets. The reduced platelet reactivity and impaired ability to contract blood clots can be a pathogenic basis for hemorrhagic complications. At the same time, the impaired platelet contractility may be pro-thrombotic, because non-compacted intravascular clots and thrombi must be more obstructive, resistant to internal fibrinolysis, and prone to rupture or embolization. We propose that this paradoxical combination of continuous immune platelet activation and their dysfunction comprise a dual and complementary mechanism underlying the propensity of SLE patients for hemostatic complications. Therefore, the signs of platelet dysfunction should be considered a risk factor for thrombotic/bleeding complications in SLE and other systemic inflammatory diseases. 

## 4. Materials and Methods

### 4.1. Clinical Characteristics of the SLE Patients Enrolled in the Study

The study was performed with the blood from 61 SLE patients not receiving aspirin on other antiplatelet drugs (see Table 3 and Table 4 for demographic, clinical, and laboratory characteristics) and 83 aspirin-free healthy donors. The control group matched the SLE patients’ group by age and gender. The study was approved by the Ethical Committee of Kazan Federal University (protocol #27 as of 28 December 2020) and performed in accordance with the Declaration of Helsinki. Informed written consent was obtained from all patients and donors. SLE patients were excluded from the study if they received anticoagulants, antiplatelet drugs, or thrombolytics within two weeks before the time of examination. Seventy percent of the patients were on corticosteroids, which might partially suppress platelet activity [62] and therefore attenuate the expression and secretion of P-selectin in the SLE patients studied, which otherwise could be even more pronounced.

### 4.2. Blood Collection and Processing

Venous blood was drawn into vacutainers containing 3.8% trisodium citrate or silica microparticles (Z serum clot activator, Greiner Bio-One, Kremsmünster, Austria). Platelet-rich plasma (PRP) was obtained from the whole citrated blood by centrifugation at 200× *g*, 10 min, and 23 °C. Platelet-poor plasma (PPP) was obtained from PRP by centrifugation at 1500× *g* for 5 min; it was aliquoted and stored at −20 °C until use. The frozen samples of PPP were thawed at 37 °C for 60 min and used within 2 h. A portion of each citrated blood sample was used for standard immunological, hematological, and biochemical tests.

### 4.3. Expression Levels of Membrane-Associated P-Selectin and Phosphatidylserines on Platelets

In dot-plots obtained with flow cytometry, platelets were labeled and gated using anti-human-CD41 antibodies conjugated with phycoerythrin (BD Bioscience, San Jose, CA, USA). Platelet surface-associated P-selectin and phosphatidylserine were determined using anti-CD62p-antibodies labeled with phycoerythrin (BD Bioscience, San Jose, CA, USA) or annexin V labeled with fluorescein-isothiocyanate (FITC) (BioLegend, San Diego, CA, USA), respectively. A 5 µL sample of PRP was diluted with 45 µL of Tyrode’s buffer (4 mM HEPES, 135 mM NaCl, 2.7 mM KCl, 2.4 mM MgCl_2_, 5.6 mM D-glucose, 3.3 mM NaH_2_PO_4_, 0.35 mg/mL bovine serum albumin, pH 7.4), mixed with 3 µL of the labeled antibodies or annexin V, and incubated for 15 min at room temperature in the dark. A 50 µL sample of diluted and labeled PRP was mixed with 350 µL of HEPES buffer (10 mM HEPES, 140 mM NaCl, 2.5 mM CaCl_2_, pH 7.4) and analyzed with FacsCalibur flow cytometer (Becton Dickinson, East Rutherford, NJ, USA). Data were processed and analyzed using FlowJo X software. The expression of platelet-associated P-selectin was measured as a fraction of P-selectin-positive platelets. The fraction of platelets expressing phosphatidylserines was determined as annexin V-FITC-positive signals in the platelet gate (Figure 1A).

### 4.4. Concentrations of Soluble P-Selectin in PPP

Soluble P-selectin (sP-selectin) was determined in PPP using the “P-Selectin (Soluble) (CD62) Human” ELISA Kit (Invitrogen, Waltham, MA, USA) according to the manufacturer’s instructions. Briefly, microwell strips were washed twice with a wash buffer followed by filling the wells with standards, controls, and 10-fold diluted PPP (100 µL final volume per well). Then, 50 µL of the horseradish peroxidase-antibody-conjugate was added to each well. After 2 h incubation at room temperature, the microwell plates were washed 3 times with the wash buffer followed by adding 100 µL of the 3,3’,5,5’-tetramethylbenzidine substrate solution to each well. After 30 min incubation at room temperature, a stop solution (100 µL) was added to each well and the color intensity at 450 nm was measured using a microplate reader (Stat Fax 2000, Awareness Technology Inc., Palm City, FL, USA).

### 4.5. Blood Clot Contraction Assay

As a measure of platelet contractility, the kinetics of blood clot contraction was studied using an original method [32] based on the optical tracking of the clot size during thrombin-induced blood clotting and clot contraction using the Thrombodynamics Analyzer System (HemaCore, Moscow, Russia) (Figure 5). Briefly, a plastic 12 × 7 × 1 mm cuvette was pre-rinsed with 4% Triton X-100 in 150 mM NaCl to prevent the adhesion of fibrin to the walls. In a separate plastic test tube, CaCl_2_ (2 mM final) and human thrombin (1 U/mL final) were added to a fresh citrated blood sample (200 µL) to initiate coagulation and activate platelets. The thrombin-activated blood (80 μL) was transferred into the measuring cuvette preheated to 37 °C. The registration of the clot size was performed every 15 s for 20 min after the addition of thrombin. The serial images of the clot were computationally quantified and converted into a kinetic curve (Figure 5), from which the following parameters were calculated: (1) the extent of the contraction, reflecting the extent of clot compaction (in percent) relative to its initial size after 20 min of registration; (2) lag time, i.e., the time during which the clot reaches 95% of its initial size; (3) the area under the kinetic curve, which reflects the amount of mechanical work on clot compression performed by the platelets; and (4) the average contraction velocity, i.e., the extent of clot compaction (%) per time unit (s). Transitions between different phases of contraction were determined by finding local maxima and minima points within the instantaneous first derivative of kinetic curves (Figure 5B). Curves were fit using a piecewise function, and the rate constants in each phase were determined. The border of the normal and impaired extent of clot contraction in this assay has been determined at the level of 41% [55].

### 4.6. Isolation of Platelets

Platelets were isolated from 1.0–1.5 mL of PRP from the blood of SLE patients or healthy donors by gel-filtration on a column filled with Sepharose 2B (GE Healthcare, Danderyd, Sweden) and equilibrated with Tyrode’s buffer (4 mM HEPES, 135 mM NaCl, 2.7 mM KCl, 2.4 mM MgCl_2_, 5.6 mM D-glucose, 3.3 mM NaH_2_PO_4_, 0.35 mg/mL bovine serum albumin, pH 7.4). Isolated platelets were collected in the void volume at a concentration of 80,000 to 240,000 platelets per 1 μL (counted in a hemocytometer at 400×).

### 4.7. Measuring Platelet Reactivity in Response to Stimulation

Isolated platelets from SLE patients or healthy donors (400,000 in 20 µL of Tyrode’s buffer) were labeled for 10 min at room temperature with anti-CD62p-antibodies conjugated with phycoerythrin (3 μL) (BD Bioscience, San Jose, CA, USA) for P-selectin detection or with human fibrinogen conjugated with Alexa Fluor-488 (5 μg/mL) (ThermoFisher Scientific, Waltham, MA, USA) for the detection of activated integrin αIIbβ3. Part of the labeled platelets was treated with 50 µM of thrombin receptor-activating hexapeptide (TRAP-6) (Bachem Americas Inc., Torrance, CA, USA) for 3 min at room temperature. The labeled platelets, treated and not treated with TRAP, were analyzed using flow cytometry.

### 4.8. Scanning Electron Microscopy of Platelets

Gel-filtered platelets were fixed in 2% glutaraldehyde in 50 mM sodium cacodylate buffer (pH 7.5), containing 150 mM NaCl, for 90 min at room temperature. The fixed platelets were layered on a carbon filter (0.1 or 0.4 μm pore size) and centrifuged at 150× *g* for 5 min. The samples were rinsed three times with the cacodylate buffer for 5 min, dehydrated in ascending concentrations of ethanol, immersed in hexamethyldisilazane, and dried overnight. A thin film of gold palladium was layered on the samples using a sputter coater Quorum Q 150T ES (Quorum, Lewes, UK). Micrographs were taken with a scanning electron microscope (Merlin, Carl Zeiss, Jena, Germany).

### 4.9. Transmission Electron Microscopy of Platelets

Gel-filtered platelets were fixed in 2% glutaraldehyde dissolved in phosphate-buffered saline (PBS) for 90 min at room temperature. The fixed platelets were centrifuged at 1000× *g* for 5 min. The pellet was washed with PBS, and the samples were post-fixed with 1% osmium tetroxide in the same buffer with addition of sucrose (25 mg/mL) for 2 h. The samples were dehydrated in ascending concentrations of ethanol, then in acetone and propylene oxide. Epon 812 was used as the embedding resin. Samples were polymerized for 3 days under increasing temperature from 37 °C to 60 °C. Sections were obtained on an LKB-III ultramicrotome (LKB, Mölndal, Sweden). The sections were contrasted with saturated aqueous solution of uranyl acetate for 10 min at 60 °C and then with an aqueous solution of lead citrate for 10 min. The samples were examined using a JEM 1200EX electron microscope (JEOL, Tokyo, Japan).

### 4.10. Statistical Analysis

Statistical and fitting analyses were performed using a GraphPad Prism 5.0 software package (GraphPad Software, San Diego, CA, USA). After the normality was assessed with the Shapiro–Wilk and D’Agostino-Pearson criteria, data arrays were analyzed using the Mann–Whitney U test or the Kruskal–Wallis test for multiple comparisons. Correlation analysis was performed using the Spearman’s rank correlation. The χ^2^-square test was used to analyze the morphological data in categorical values. The significance level was 95% (*p* < 0.05).

## Figures and Tables

**Table 1:** Fraction of platelets with background expression of phosphatidylserine or P-selectin (**a**), levels of sP-selectin (**b**), and parameters of clot contraction kinetics (**c**,**d**) in clots made from the blood of SLE patients and healthy subjects.

	SLE Patients		Control		
	*n*	Median (IQR)	*n*	Median (IQR)	*p* Value ^1^
**(a) Phosphatidylserine and P-selectin expression**					
Phosphatidylserine-positive unstimulated platelets, %	34	2.2 (1.1–7.2)	22	1.0 (0.5–1.9)	0.002
P-selectin-positive unstimulated platelets, %	56	4.5 (2.9; 8.2)	14	3.5 (2.2; 4.1)	0.032
**(b) soluble P-selectin secretion**					
sP-selectin in blood plasma, ng/mL	53	51 (38; 75)	15	34 (29; 39)	<0.001
**(c) Platelet contractility, parameters of blood clot contraction**					
Extent of blood clot contraction, %	55	35.3 (25.8; 45.8)	40	48 (43; 52)	<0.001
Lag time of blood clot contraction, sec	55	150 (108; 236)	40	75 (45; 120)	<0.001
Area over the kinetic curve of blood clot contraction, a.u.	55	259 (176; 317)	40	380 (316; 447)	<0.001
Average velocity of blood clot contraction, (%/sec) × 10^−3^	55	29 (20; 37)	40	38(35; 42)	<0.001
**(d) Kinetic phase analysis of clot contraction**					
Rate constant for phase 1, s^−1^	55	0.012	40	0.014	n.s.
Rate constant for phase 2, %/s	55	0.032	40	0.047	<0.0001
Rate constant for phase 3, s^−1^	55	0.0005	40	0.0017	<0.0001

^1^ Mann–Whitney test. In (a), (b), and (c), the results are presented as a median (IQR). n.s. means not significant.

**Table 2:** Spearman’s correlation coefficients for the fractions of phosphatidylserine-expressing platelets (**a**), P-selectin-expressing platelets (**b**), and soluble P-selectin levels (**c**) versus the markers of immune inflammation and parameters of clot contraction in all the SLE patients studied and in two patient subgroups, i.e., patients with and without the antiphospholipid syndrome (APS).

**(a) Phosphatidylserine-expressing platelets vs. the markers of immune inflammation and clot contraction**				
	**SLEDAI**	**Leukocyte Count**		**Lag Time of Clot Contraction**
Phosphatidylserine-positive platelets in all SLE patients	r = 0.48 *p* = 0.012	r = −0.35 *p* = 0.043		r = −0.39 *p* = 0.029
Phosphatidylserine-positive platelets in SLE patients with APS	r = 0.84 *p* = 0.029	n.s.		n.s.
Phosphatidylserine-positive platelets in SLE patients without APS	n.s.	n.s.		n.s.
**(b) P-selectin-expressing platelets vs. the markers of immune inflammation and clot contraction**				
	**IgG**	**IgA**	**Anti-dsDNA Abs**	**Extent of Clot Contraction**
Platelet-associated P-selectin in all SLE patients	r = 0.33 *p* = 0.031	n.s.	r = 0.46 *p* = 0.013	r = 0.31 *p* = 0.029
Platelet-associated P-selectin in SLE patients with APS	r = 0.67 *p* = 0.039	r = 0.69 *p* = 0.032	n.s.	n.s.
Platelet-associated P-selectin in SLE patients without APS	n.s.	n.s.	n.s.	r = 0.35 *p* = 0.02
**(c) sP-selectin vs. the markers of immune inflammation**				
	**IgM**	**IgA**		**Anti-dsDNA Abs**
sP-selectin levels in all SLE patients	r = 0.47 *p* = 0.003	r = 0.35 *p* = 0.031		r = 0.43 *p* = 0.030
sP-selectin levels in SLE patients with APS	r = 0.74 *p* = 0.045	r = 0.67 *p* = 0.039		r = 0.82 *p* = 0.034
sP-selectin levels in SLE patients without APS	n.s.	n.s.		n.s.

n.s. = not significant.

**Table 3:** Demographic and clinical characteristics of SLE patients enrolled in this study.

Demographic and Clinical Characteristics		SLE Patients (*n* = 61)
Sex, male/female, *n*		3 (5%)/58 (95%)
Age (years), median; min-max		40; 19–64
Duration of SLE * (years), median; IQR		4; 1–8.5
Patients receiving immunosuppressive treatment *, *n*		47 (77%)
SLEDAI *, median; IQR		4; 2–10
Antiphospholipid syndrome, *n*		12 (20%)
Renal features, *n*	Lupus nephritis	40 (66%)
	Hematuria	6 (10%)
	Proteinuria	11 (18%)
Musculoskeletal features, *n*		24 (39%)
Mucocutaneous features, *n*		29 (48%)
Cardiovascular features, *n*		17 (28%)
Neurological manifestations, *n*		5 (8%)
Pulmonary manifestations, *n*		7 (11%)
History of thrombosis, *n*		8 (13%)
Obstetric manifestations (miscarriages/stillbirths), *n*		5 (8%)
History of hematologic features, *n*	Leukopenia	20 (33%)
	Anemia	29 (48%)
	Thrombocytopenia	8 (13%)

* At the time of examination.

**Table 4:** Parameters of blood tests in SLE patients enrolled in the study.

Blood Tests (Normal Ranges Are Shown in Parentheses)	SLE Patients (*n* = 61)
*Immunologic parameters*	Median (IQR)
Anti-dsDNA antibodies (<10), IU/mL	22 (7–200)
Anti-cardiolipin antibodies (<10), IU/mL	1.8 (1.4–5.1)
IgA (1.1–3.5), mg/mL	2.0 (1.5–3.8)
IgM (0.7–2.5), mg/mL	1.3 (0.9–2.6)
IgG (6.7–16.5), mg/mL	11.3 (7.5–17.7)
Total complement activity (31–60) U/mL	31 (28–36)
Circulating immune complexes (<50), U/mL	102 (62–128)
Prothrombin time (9.2–12.2), s	11.0 (10.3–11.8)
Fibrinogen (2–4), g/L	3.9 (3.5–4.4)
INR (0.9–1.5)	0.9 (0.88–1)
*Hematologic parameters*	
Platelets (140–380), × 10^9^/L	230 (188–286)
Red blood cells (3.9–4.7), × 10^12^/L	4.2 (3.8–4.4)
Leukocytes (4–9), × 10^9^/L	6.2 (4.8–7.9)
Neutrophils (40–70), %	63 (53–71)
Lymphocytes (19–37), %	28 (21–35)
Monocytes (3–9), %	7 (5.1–9.8)
Hemoglobin (12–14), g/dL	11.3 (9–12.5)
Erythrocyte sedimentation rate (3–16), mm/h	17 (8.5–27.5)
*Biochemical parameters*	
Glucose (3.8–6.1), Mmol/L	4.6 (4.1–4.9)
ALT (<45), U/L	15.5 (11.3–19.1)
AST (<35), U/L	13.6 (11–17)
Creatinine (45–100), μmol/L	60 (50–79)
Cholesterol (2.2–5.7), mmol/L	5.5 (4.5–6.1)

## Abbreviations

APS	antiphospholipid syndrome
ALT	alanine transaminase
AST	aspartate transaminase
FITC	fluorescein isothiocyanate
IL	interleukin
INR	international normalized ratio
PE	phycoerythrin
PPP	platelet-poor plasma
PRP	platelet rich plasma
PSGL-1	P-selectin glycoprotein ligand-1
SLE	systemic lupus erythematosus
SLEDAI	Systemic Lupus Erythematosus Disease Activity Index
sP-selectin	soluble P-selectin
TRAP	thrombin receptor-activating peptide-6
